# Derangement of a Factor Upstream of RARα Triggers the Repression of a Pleiotropic Epigenetic Network

**DOI:** 10.1371/journal.pone.0004305

**Published:** 2009-02-04

**Authors:** Francesca Corlazzoli, Stefano Rossetti, Gaia Bistulfi, Mingqiang Ren, Nicoletta Sacchi

**Affiliations:** Cancer Genetics Program, Roswell Park Cancer Institute, Buffalo, New York, United States of America; Baylor College of Medicine, United States of America

## Abstract

**Background:**

Chromatin adapts and responds to extrinsic and intrinsic cues. We hypothesize that inheritable aberrant chromatin states in cancer and aging are caused by genetic/environmental factors. In previous studies we demonstrated that either genetic mutations, or loss, of retinoic acid receptor alpha (RARα), can impair the integration of the retinoic acid (RA) signal at the chromatin of RA-responsive genes downstream of RARα, and can lead to aberrant repressive chromatin states marked by epigenetic modifications. In this study we tested whether the mere interference with the availability of RA signal at RARα, in cells with an otherwise functional RARα, can also induce epigenetic repression at RA-responsive genes downstream of RARα.

**Methodology/Principal Findings:**

To hamper the availability of RA at RARα in untransformed human mammary epithelial cells, we targeted the cellular RA-binding protein 2 (CRABP2), which transports RA from the cytoplasm onto the nuclear RARs. Stable ectopic expression of a CRABP2 mutant unable to enter the nucleus, as well as stable knock down of endogenous CRABP2, led to the coordinated transcriptional repression of a few RA-responsive genes downstream of RARα. The chromatin at these genes acquired an exacerbated repressed state, or state “of no return”. This aberrant state is unresponsive to RA, and therefore differs from the physiologically repressed, yet “poised” state, which is responsive to RA. Consistent with development of homozygosis for epigenetically repressed loci, a significant proportion of cells with a defective CRABP2-mediated RA transport developed heritable phenotypes indicative of loss of function.

**Conclusion/Significance:**

Derangement/lack of a critical factor necessary for RARα function induces epigenetic repression of a RA-regulated gene network downstream of RARα, with major pleiotropic biological outcomes.

## Introduction

Retinoic acid (RA), the bioactive derivative of retinol, is a signal fundamental for developmental and cellular processes, whose intracellular physiological level is tightly regulated by a complex metabolic pathway involving both RA synthesis and RA catabolism [1], [2]. RA exerts its biological action mainly by binding and activating specialized transcription factors, the RA-receptors (RARs) [3]. When RA is channeled onto the retinoic acid receptor alpha (RARα) in the nucleus, it can rapidly induce transcription of RARα-target genes containing a RA-responsive element (RARE). Specifically, RA binding to RARα triggers both the dissociation of corepressors proteins, and the recruitment of coactivators and histone modifying enzymes that enable chromatin conformation changes compatible with the access and action of RNA polymerase II [4], [5].

The temporal dynamics of the cascade of events following RA-RARα-mediated chromatin activation has been mostly derived from studies on the prototypic direct RARα-target gene *RARβ2*. Once expressed in response to RA, RARβ2 sustains its own transcription by binding to its own promoter [6], and subsequently activates the chromatin of other downstream RA-responsive direct target genes [7], [8]. In the absence of RA, *RARβ2* chromatin reaches a repressed state, which is however poised for transcription [4], [5].

Previously, we demonstrated that when RA signal cannot be integrated at RARα, because RARα is either not expressed, or has acquired genetic mutations that make it non-functional, the chromatin associated with *RARβ2* falls into an aberrant exacerbated state of repression, which is unresponsive to RA [9]. Moreover, by using different cell systems, we demonstrated that the impaired integration of RA signal at a mutant RARα induces a repression wave that is propagated, in a domino fashion, from *RARβ2* to targets downstream of RARβ2. Specifically, by using mouse embryocarcinoma cells, we found that a dominant negative RARα mutant creates a concerted repression of both *RARβ2* and its direct target *CYP26A1*, encoding the cytochrome P450 RA-specific hydrolase, which acts as a neuronal differentiation switch in these cells [8], [10]. In an independent study using human mammary epithelial cells, we demonstrated that inhibition of RARα function with various genetic strategies triggers the concerted repression of both *RARβ2* and another target downstream of RARβ2, *CRBP1*, encoding the cellular retinol binding protein 1, which is pivotal for breast epithelial cell acinar morphogenesis [7].

Based on the observation that the *RARβ2* chromatin can also be found aberrantly repressed in RARα-positive cancer cells [11], we hypothesized that lack/derangement of upstream factors capable of affecting RARα function is sufficient to induce aberrant chromatin repression at *RARβ2* and its downstream targets.

In the present study we show that the derangement of the cellular RA binding protein 2 (CRABP2), critical for the transport of RA from the cytoplasm to the RARs in the nucleus [12], can indeed trigger a long-distance chromatin repression effect at *loci* of an entire RARα-regulated epigenetic network. We found that, not only the knock down of endogenous CRABP2 by RNAi, but simply the mere interference of RA transport into the nucleus, achieved by expressing a dominant negative CRABP2 mutant unable to enter the nucleus [13], can initiate the wave of aberrant repression at the chromatin of multiple RA-responsive genes. The wave of repression involves first *RARβ2*, thus affecting cell growth, and next branches downstream, to involve genes that control both RA metabolism/homeostasis and morphogenesis.

In conclusion, interference with RA transport at RARα into the nucleus is sufficient to induce coordinated, heritable, chromatin repression at multiple loci of a RA-responsive gene network downstream of RARα, with pleiotropic biological outcomes.

## Results

### Interference with RA transport into the nucleus is sufficient to induce transcriptional repression of genes downstream of RARα

**Figure 1:**
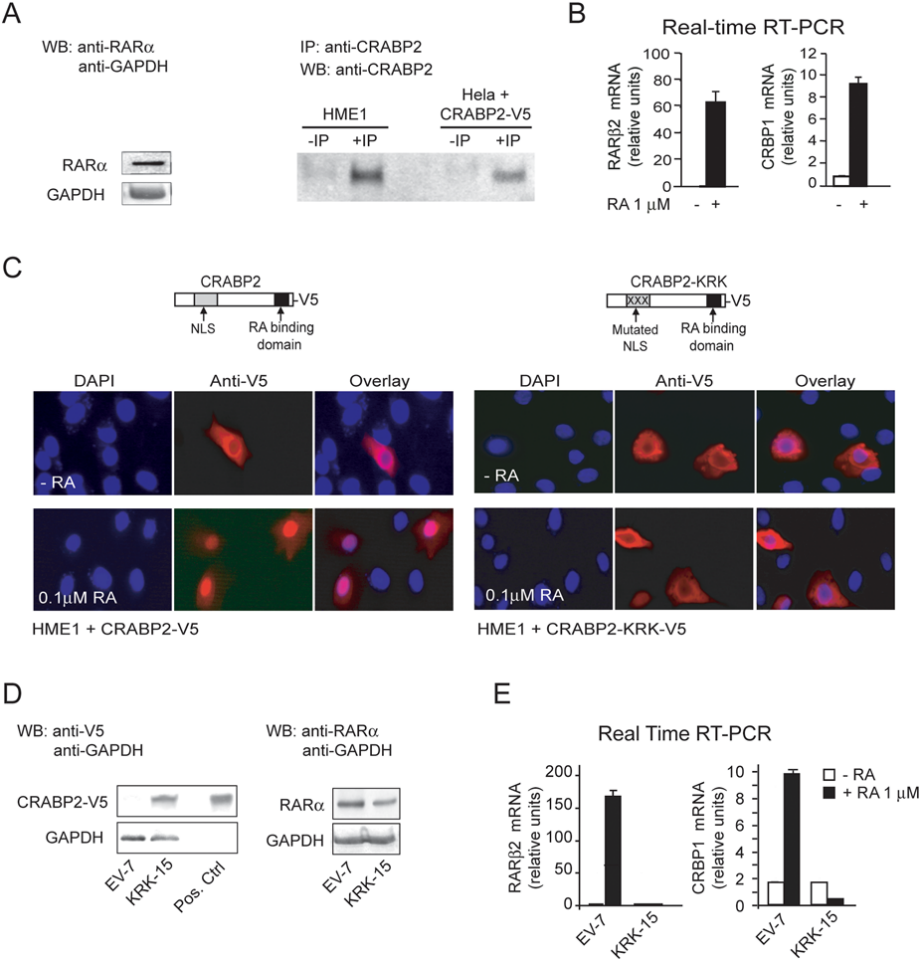
Interference with RA transport into the nucleus is sufficient to induce transcriptional repression of genes downstream of RARα. (A) WB analysis showing that HME1 cells express RARα (left). Immunoprecipitation (IP) followed by WB showing that HME1 express CRABP2. IP of Hela cells transiently transfected with CRABP2-V5 served as positive control (right). (B) Transcriptional activation of two RA-responsive genes, *RARβ2* and *CRBP1*, in response to RA (72 h) demonstrates a functional RA-RARα signaling in HME1 cells. (C) Transient HME1 transfection with wild type CRABP2-V5, followed by immunocytochemistry with anti-V5 (red) and DAPI nuclear staining (blue), shows that exogenous CRABP2-V5 can translocate from the cytoplasm into the nucleus after treatment with 0.1 μM RA for 30 min. (left). In contrast, exogenous CRABP2-KRK-V5 mutant carrying a mutated nuclear localization signal (NLS) is not able to enter the nucleus under the same conditions (right). (D) WB analysis showing the expression of the CRABP2-KRK-V5 protein in the HME1-derived clone KRK-15, but not in the HME1 control clone EV7. *In vitro* transcribed and translated CRABP2-KRK-V5 was used as a positive control (left). Both KRK-15 and EV-7 cells express RARα (right). (E) Both *RARβ2* and *CRBP1* transcription are significantly less inducible by RA (72 h) in KRK-15 cells relative to the control EV7 cells.

RARα activation requires the transport of RA to RARα in the nucleus by CRABP2 [12], [14], [15]. HME1 cells, which express both RARα (Fig. 1A left) and CRABP2 (Fig. 1A, right), can properly integrate RA signal through RARα, as demonstrated by the transcriptional activation of two prototypic RA-responsive genes, *RARβ2,* a downstream RARα target, and *CRBP1*, a downstream RARβ2 target (Fig. 1B, left and right).

To transport RA into the nucleus, CRABP2 requires a specific nuclear localization signal (NLS) [13]. A mutant CRABP2-KRK protein, which was shown to bind RA with affinity similar to the one of the wild type CRABP2 protein, cannot transport RA into the nucleus due to critical mutations in the NLS [13]. Indeed, by using immunocytochemistry, we found that the V5-tagged CRABP2-KRK protein, transiently expressed in HME1 cells, differently from the wild type CRABP2-V5 protein, is not able to enter the nucleus after addition of RA (0.1 μM, 30 minutes) (Fig. 1C).

Next, we tested whether RA transport into the nucleus is hampered in CRABP2-KRK-positive cells. Stable expression of the CRABP2-KRK-V5 protein in HME1 cells (shown for the KRK-15 clone in Fig. 1D, left), while not affecting the expression of endogenous RARα relative to the control clone EV7 (Fig. 1D, right), clearly exerts a dominant negative effect over the endogenous CRABP2. This conclusion is based on the observation that RA-induced transcriptional activation of both *RARβ2* and *CRBP1* is reduced in the KRK-15 clone relative to the control clone EV7 (Fig. 1E, left and right). Thus, targeting CRABP2 function prevents RARα function and affects, in a negative and irreversible fashion, the transcriptional status of RA-responsive genes downstream of RARα.

### Evidence of chromatin repression at RA-responsive genes downstream of RARα consequent to CRABP2 knock-down

To test whether targeting endogenous CRABP2 in HME1 cells can indeed induce heritable aberrant repression of the chromatin at both *RARβ2* and *CRBP1*, we knocked down CRABP2 by stable RNA interference with either one of two *CRABP2*-targeting shRNA sequences, CRABP2-A and CRABP2-C (Fig. S1A). A scrambled (mock) shRNA sequence, which should not recognize any human mRNA, was used as a control (Fig. S1A). Only the shRNAs sequences directed against CRABP2 were shown to efficiently decrease exogenous CRABP2 protein expression (Fig. S1B).

**Figure 2:**
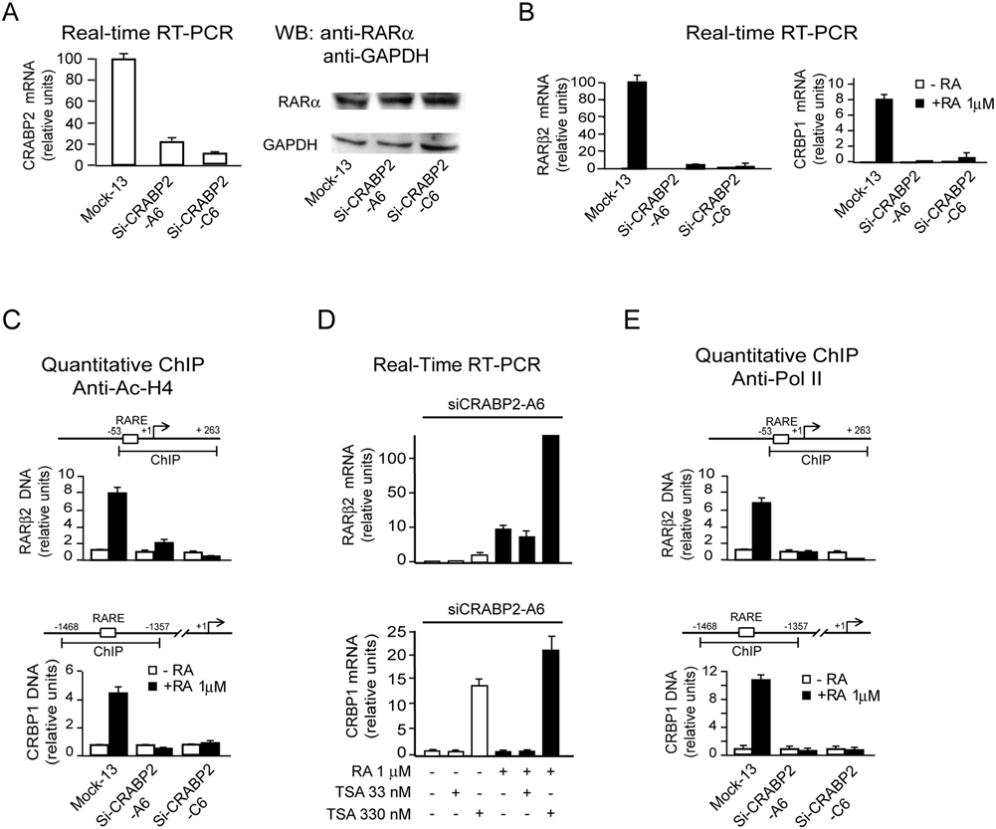
Evidence of chromatin repression at RA-responsive genes downstream of RARα consequent to CRABP2 knock-down. (A) The HME1-derived stable clones Si-CRABP2-A6 and Si-CRABP2-C6, carrying two distinct CRABP2-targeting shRNA sequences (CARBP2-A and CRABP2-C, respectively), display a significant decrease in *CRABP2* transcript relative to the control clone Mock-13 (left). The level of RARα expression is similar in Si-CRABP2-A6, Si-CRABP2-C6 and the control clone Mock13 (right). (B) Both *RARβ2* (left) and *CRBP1* (right) are significantly less inducible by RA (72 h) in Si-CRABP2-A6 and Si-CRABP2-C6 clones relative to the control Mock13 clone. (C) qChIP analysis with anti-acetyl histone H4 (Ac-H4) showing that *RARβ2* (top) and *CRBP1* (bottom) chromatin of both Si-CRABP2-A6 and Si-CRABP2-C6 is marked by H4 hypoacetylation at the RARE-containing regulatory regions relative to the control clone Mock-13. (D) RA-induced *RARβ2* (top) and *CRBP1* (bottom) transcription can be restored in Si-CRABP2-A6 cells by treatment with the HDAC inhibitor TSA. (E) qChIP with anti-Polymerase II (Pol II) showing decreased occupancy of Pol II at the RARE-containing regions of both *RARβ2* (top) and *CRBP1* (bottom) in Si-CRABP2-A6 and Si-CRABP2-C6.

We further tested two CRABP2 knock down clones, Si-CRABP2-A6, carrying the CRABP2-A sequence, and Si-CRABP2-C6, carrying the CRABP2-C sequence, along with the control clone Mock13, carrying the scrambled sequence (Fig. S1C). Both Si-CRABP2-A6 and Si-CRABP2-C6 displayed a significant decrease of the CRABP2 transcript (Fig. 2A, left), while they still expressed the RARα receptor (Fig. 2A, right). RA failed to activate the transcription of both *RARβ2* and *CRBP1* in both knock down clones (Fig. 2B, left and right).

Moreover, ChIP analysis with anti-acetylated histone H4 (Ac-H4) showed significant hypoacetylation, which remained unresponsive to RA, of the chromatin regions encompassing either the *RARβ2*-RARE or the *CRBP1*-RARE (Fig. 2C, top and bottom). Apparently, the chromatin at both *RARβ2* and *CRBP1* was converted from a state poised for transcription to an exacerbated repressed state unresponsive to RA, which could be reverted only by treatment with the HDAC inhibitor Trichostatin A (TSA) (Fig. 2D, top and bottom). This conclusion was consistent with ChIP analysis with an anti-RNA Polymerase II (Pol II) antibody showing that both *RARβ2*-RARE and *CRBP1*-RARE chromatin regions have become inaccessible to RNA Polymerase II (Fig. 2E, top and bottom).

Thus, as a consequence of CRABP2 knock down, the chromatin of two *loci* downstream of RARα has acquired a repressed “state of no return”, unresponsive to RA. This state, non-permissive for transcription, differs from the poised state, responsive to RA, which is permissive for transcription.

### Hampering CRABP2 function in HME1 cells leads to biological phenotypes that reflect homozygosis for epigenetically silent *RARβ2* and *CRBP1* alleles

**Figure 3:**
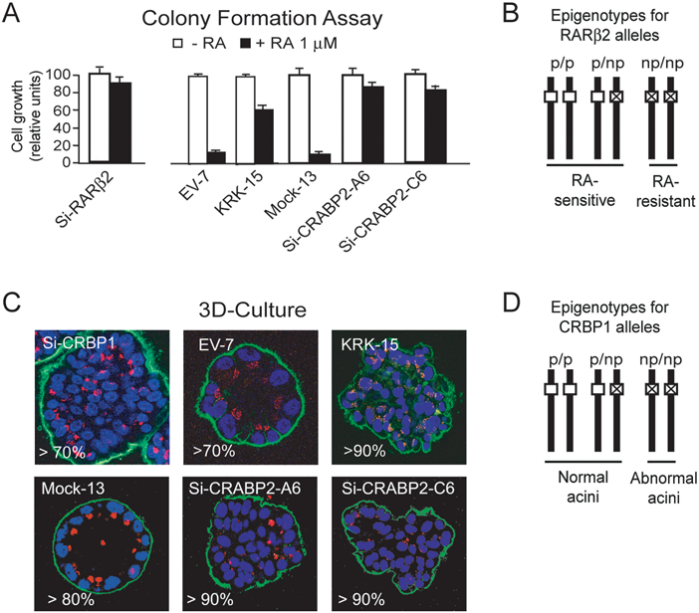
Hampering CRABP2 function in HME1 cells leads to biological phenotypes that reflect homozygosis for epigenetically silent *RARβ2* and *CRBP1* alleles. (A) HME1 cells knocked down for RARβ2 (Si-RARβ2) develop resistance to RA growth-inhibitory action (left). The HME1 clones KRK-15, Si-CRABP2-A6 and Si-CRABP2-C6, with impaired CRABP2 function, show a significantly higher fraction of RA-resistant cells than the cognate control clones EV7 and Mock-13 (right). (B) Scheme showing that RA-resistance is expected only in cells homozygous for *RARβ2* alleles non-permissive for transcription (np/np), but not in cells either homozygous for permissive *RARβ2* alleles (p/p), or heterozygous for permissive and non-permissive *RARβ2* alleles (p/np). (C) HME1 cells knocked down for CRBP1 (Si-CRBP1, top left) are unable to form hollow, polarized acini in three-dimensional (3D) culture, as shown by confocal fluorescence microscopy (nuclei are visualized in blue, integrin in green, and the Golgi apparatus in red). HME1 clones with an impaired CRABP2 function (KRK15, Si-CRABP2-A6 and Si-CRABP2-C6) are also unable of proper acinar morphogenesis. (D) Scheme showing that impaired acinar morphogenesis is expected only in cells that have developed homozygosis for non-permissive *CRBP1* alleles.

We previously demonstrated that knock down of the tumor suppressor *RARβ2* in HME1 cells confers resistance to RA-induced growth inhibition [7] (Fig. 3A, left). Analysis of RA-resistance by colony formation in HME1-derived clones with either ectopic expression of CRABP2-KRK (CRABP2-KRK15), or CRABP2 knock down (Si-CRABP2-A6 and Si-CRABP2-C6) (Fig. 3A, right) clearly indicated loss of *RARβ2* function. RA-resistance is expected only in association with homozygous repression of the chromatin at *RARβ2* alleles, which are consequently non permissive (np) for transcription (Fig. 3B). Similarly, we previously demonstrated that *CRBP1* knock down in HME1 cells hampers acinar morphogenesis in 3D culture [7] (Fig. 3C). We observed aberrant acinar morphology also in HME1-derived clones with either ectopic expression of CRABP2-KRK (CRABP2-KRK15) or CRABP2 knock down (Si-CRABP2-A6 and Si-CRABP2-C6) (Fig. 3C), thus indicating loss of CRBP1 function. Loss of proper acinar morphogenesis is expected only in association with homozygous repression of the chromatin at *CRBP1* alleles, which are consequently non permissive (np) for transcription (Fig. 3D).

Interference with CRABP2 function apparently induces loss of both RA-induced growth inhibition and 3D-acinar morphogenesis in a significant fraction of cells, strongly suggesting the occurrence of heritable homozygous epigenetic silencing at both *RARβ2* and *CRBP1 loci*.

### Evidence of CpG hypermethylation corroborates the occurrence of heritable epigenetic silencing at both *RARβ2* and *CRBP1* consequent to deranged CRABP2 function

**Figure 4:**
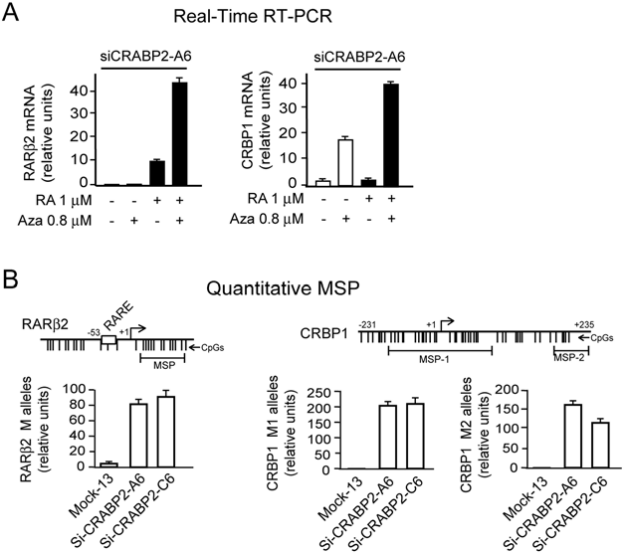
Evidence of CpG hypermethylation corroborates the occurrence of epigenetic silencing at both *RARβ2* and *CRBP1* consequent to impaired CRABP2 function. (A) Treatment with the demethylating agent 5-Aza can restore RA-induced transcription from repressed *RARβ2* and *CRBP1* chromatin in Si-CRABP2-A6 cells (left and right, respectively). (B) Quantitative MSP detecting methylated (M) alleles shows hypermethylation of *RARβ2* (left) and *CRBP1* (right) CpG-rich regulatory regions in Si-CRABP2-A6 and Si-CRABP2-C6 clones.

DNA hypermethylation is an epigenetic and heritable modification. For this reason, we tested for DNA hypermethylation at *RARβ2* and *CRBP1* in HME1 cells with deranged CRABP2 function. First, we found that treatment of Si-CRABP2-A6 cells with the demethylating agent 5-aza-2′-deoxycitidine (5-Aza) could significantly restore RA-induced *RARβ2* and *CRBP1* transcription (Fig. 4A, left and right). Then, we tested by quantitative methylation specific PCR (qMSP) whether *RARβ2* and *CRBP1* regulatory regions in the CRABP2 knock down clones were indeed marked by DNA hypermethylation. For the detection of *RARβ2* methylated (M) alleles, we used primers previously shown to recognize the *RARβ2* methylation epicenter [9], while for *CRBP1* we used primers recognizing the two regions, M1 and M2, within the *CRBP1* CpG island that we demonstrated previously to be the first undergoing aberrant DNA methylation in cells with an impaired RARα signaling [7]. This analysis clearly shows that CRABP2 knock down clones A6 and C6 have significantly more *RARβ2* and *CRBP1* methylated (M) alleles relative to the control clone Mock13 (Fig. 4B, left and right). The finding that *RARβ2* and *CRBP1* silencing is associated with DNA hypermethylation, a well-established hallmark of aberrantly repressed chromatin, further reinforces our conclusion that the repressed state of *RARβ2* and *CRBP1* chromatin in cells with deranged CRABP2 function is heritable, and therefore epigenetic.

### Derangement of CRABP2 function exerts a chromatin repression effect branching downstream of RARβ2

We previously demonstrated in a mouse embryonic carcinoma cell model that an endogenous dominant negative RARα mutant, lacking part of the E domain harboring the RA-binding domain, induced concerted epigenetic repression of both *RARβ2* and *CYP26A1,* encoding for a RA hydrolase involved in RA catabolism [8]. We reproduced this finding also in human cells carrying an exogenous dominant negative RARα mutant lacking the RA-binding domain (Fig. S2). Here we show that hampering CRABP2 function leads to significant *CYP26A1* chromatin repression also in HME1 cells.

**Figure 5:**
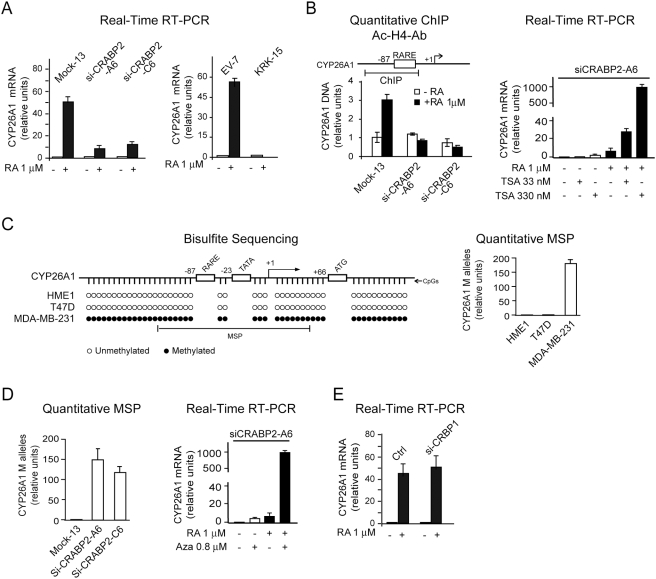
Derangement of CRABP2 function exerts a chromatin repression effect branching downstream of RARβ2. (A) RA-induced transcription of *CYP26A1* is significantly downregulated in both HME1 cells knocked down for CRABP2 (Si-CRABP2-A6 and Si-CRABP2-C6 clones, left), and HME1 cells carrying the CRABP2-KRK mutant (KRK-15 clone, right) relative to control cells (Mock 13 and EV7 clones, respectively). (B) qChIP analysis with anti-acetyl histone H4 showing that *CYP26A1* chromatin in Si-CRABP2-A6 and Si-CRABP2-C6 clones is marked by a significant H4 hypoacetylation of a region encompassing the *CYP26A1* proximal RARE (left). RA-induced *CYP16A1* transcription can be restored in Si-CRABP2-A6 by treatment with the HDAC inhibitor TSA for 72 h (right). (C) Bisulfite sequencing showing that HME1, like the CYP26A1-positive cell line T47D, is unmethylated in the proximal RARE-containing CpG island, while the CYP26A1-negative cell line MDA-MB-231 is fully methylated (left). Quantitative MSP with primers recognizing only methylated (M) alleles can detect methylation in MDA-MB-231, but not in T47D or HME1 cells (right). (D) Quantitative MSP analysis showing hypermethylation of *CYP2A1* proximal CpG island in Si-CRABP2-A6 and Si-CRABP2-C6 clones (left). RA-induced *CYP26A1* transcription can be efficiently restored in Si-CRABP2-A6 cells by treatment with the demethylating agent 5-Aza (right). (E) *CYP26A1* transcription can still be induced by RA in HME1 cells knock down for CRBP1 (Si-CRBP1). Thus, *CYP26A1* epigenetic downregulation is not consequent to *CRBP1* epigenetic silencing.

First, we found that impairment of CRABP2 function in HME1 cells by either CRABP2 knock down, or expression of the mutant CRABP2-KRK, leads to significant downregulation of RA-induced *CYP26A1* transcription (Fig. 5A, left and right, respectively). *CYP26A1* transcription is driven by a promoter region containing a proximal RARE at −87 and seems to be enhanced by an upstream region containing a distal RARE at −1973 [16], [17]. ChIP analysis with anti-acetyl histone H4 shows that *CYP26A1* downregulation in Si-CRABP2-A6 and Si-CRABP2-C6 clones is marked by histone deacetylation, which is unresponsive to RA, both in the region containing the distal RARE (data not shown) and in the region containing the proximal RARE (Fig. 5B, left). Consistently, treatment with the HDAC inhibitor TSA could efficiently restore RA-induced *CYP26A1* transcription in CRABP2 knock down clones (shown here for Si-CRABP2-A6 in Fig. 5B, right).

Second, we tested whether the *CYP26A1* repressed chromatin state, consequent to CRABP2 knock down, was also marked by DNA hypermethylation. By *in silico* analysis of the 5′ regulatory regions of human *CYP26A1,* we identified two canonical CpG islands: one encompassing the distal RARE, and one encompassing the proximal RARE (Fig. S3). Bisulfite sequencing of these two regions showed that the proximal CpG island is fully methylated in the CYP26A1-negative cell line MDA-MB-231, while it is fully unmethylated in two CYP26A1-positive cell lines, T47D and HME1 (Fig. 5C, left). In contrast, the methylation status of the distal CpG island did not show any significant difference between HME1 and MDA-MB-231 (data not shown). Therefore, we focused our analysis on the proximal CpG island. By using qMSP with primers able to discriminate between the different methylation status of the control cell lines HME1, T47D and MDA-MB-231 (Fig. 5C, right), we found that the CRABP2 knock down clones have significantly more *CYP26A1* methylated (M) alleles relative to the control clone Mock13 (Fig. 5D, left). Consistently, treatment with the demethylating agent 5-Aza could significantly restore RA-induced *CYP26A1* transcription in CRABP2 knock down cells (shown here for Si-CRABP2-A6 in Fig. 5D, right).

**Figure 6:**
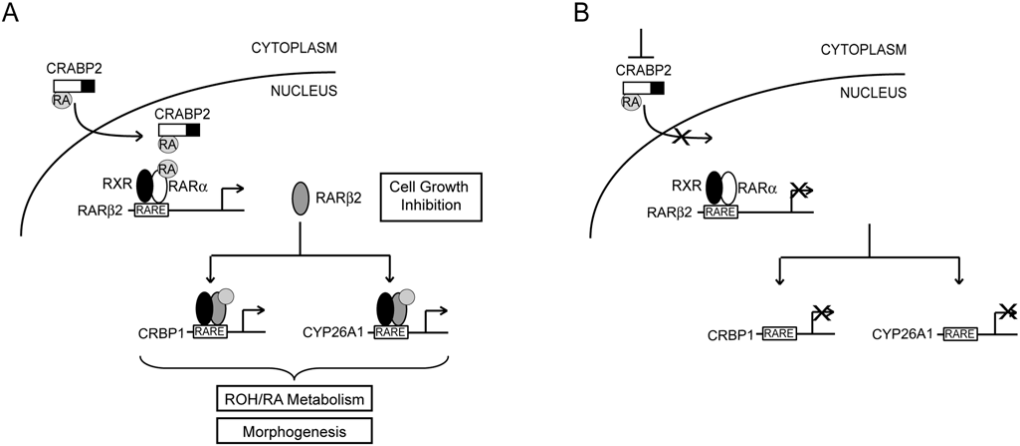
Epigenetic repression of a pleiotropic gene network as a consequence of a defective RA transport onto RARα. (A) RA transport onto RARα by CRABP2 enables the transcriptional activation of a RA-responsive gene network involved in retinol (ROH)-RA metabolism, control of cell growth, and morphogenesis. (B) Interference with CRABP2-mediated RA transport onto RARα leads to epigenetic repression of this gene network, with pleiotropic biological outcome.

Finally, we asked whether *CYP26A1* epigenetic downregulation is consequent to, or concomitant with, the epigenetic downregulation of *CRBP1,* the other RARβ2 target. We found that *CYP26A1* transcription is still RA-inducible in HME1 cells knocked down for CRBP1 (Si-CRBP1) (Fig. 5E). Thus, *CYP26A1* transcriptional downregulation, induced by hampering CRABP2 function, is consequent to a “long distance” repression effect, branching downstream of RARβ2, and involving both *CRBP1* and *CYP26A1* chromatin (Fig. 6).

## Discussion

In different cell systems, and using different mechanistic approaches, we previously demonstrated that an impaired RARα signalling, due to derangement/loss of RARα itself, confers an exacerbated repressed chromatin state, marked by repressive epigenetic changes at several RA-responsive genes downstream of RARα [7]–[9]. This study shows that hampering CRABP2, a factor critical for RA transport onto nuclear RARα, in cells with a functionally intact RARα, also leads to epigenetic repression of RA-responsive genes downstream of RARα, with heritable biological outcomes.

We provide evidence that derangement of CRABP2 function is sufficient to trigger the coordinated repression of the RARα direct target *RARβ2*, and two RARβ2 downstream targets, *CRBP1* and *CYP26A1*. Specifically, in HME1 cells with functional RARα, we observed that not only the silencing of endogenous *CRABP2*, but the mere interference with CRABP2-mediated RA-transport into the nucleus, achieved by expressing the CRABP2-KRK protein with a mutated nuclear localization signal, induces heritable epigenetic changes at genes of a RA-responsive gene network downstream of RARα (Fig. 6).

Apparently, the abrogation of RARα function, be it due to *RAR*α silencing/genetic mutations, or derangement of a factor upstream of RARα (e.g. CRABP2), results in the conversion of the chromatin of RARα-regulated genes from an inactive, yet poised, state permissive for transcription into an exacerbated repressed state that is non permissive for transcription. We refer to the latter state as the “state of no return”, because it is marked by repressive epigenetic modifications, which remain unresponsive to RA [9]. This exacerbated, repressed state is distinct from the physiological repressed poised state, which is still responsive to RA. We still do not know what molecular mechanism(s) is capable of “invoking” the recruitment of chromatin repressive activities at RA-responsive genes downstream of RARα, once RARα function is impaired.

As a result of derangement of CRABP2 function, and consequent impairment of RARα function, we found evidence that cells develop homozygosis for epigenetically silent genes that are either RA-receptors (RARβ2) or RA-responsive genes involved in both RA metabolism and morphogenesis (CRBP1 and CYP26A1). Specifically, we demonstrated that the homozygous epigenotypes for these repressed genes are heritable based on the analysis of biological and morphological phenotypes in HME1 cells either carrying the dominant negative mutant CRABP2 protein, or knocked down for *CRABP2*. Even when RARα was still expressed, we observed in a significant fraction of cells both RA resistance, indicative of loss of RARβ2 function, and aberrant acinar morphogenesis, indicative of loss of CRBP1 function. The RA-resistant phenotype and the aberrant acinar morphology is expected to reflect only epigenotypes homozygous for repressed, non-permissive *RARβ2* and *CRBP1* alleles, respectively. Consistently, in the same cells, we found evidence of aberrant CpG methylation, an epigenetic hallmark of repressed chromatin, at both *RARβ2* and *CRBP1*. The repressive repercussion due to derangement of CRABP2 function affects also the chromatin of *CYP26A1*, another RA-responsive gene downstream of both RARα and RARβ2. Downregulation of *CYP26A1* transcription is marked by both hypoacetylation unresponsive to RA and hypermethylation of the CpG island containing the proximal *CYP26A1* RARE. Apparently, RARs and genes of the RA metabolism (CRBP1 and CYP26A1), are part of the same network. This RARα-regulated gene network is clearly implicated also in cell growth and cell morphogenesis. Further, this gene network can undergo concerted epigenetic repression as a consequence of derangement of factor(s) capable of interfering with RARα function.

In conclusion, this study reinforces the supposition that epigenetic repression in cancer cells may result from an ordered, rather than random, re-programming of the chromatin in response to intrinsic and extrinsic cues; which mirrors the order that underlies development [18].

## Materials and Methods

### Cell cultures

#### Cells

The human immortalized, non-transformed breast epithelial cell strain hTERT-HME1, here referred to as HME1, was grown in Mammary Epithelial Growth Medium (MEGM) plus bovine pituitary extract as per manufacturer's instructions (Lonza, Walkersville, MD). The HME1-derived clones knock down for RARβ2 and CRBP1 have been described in [7]. The human breast cancer cell lines T47D and MDA-MB-231 (ATCC, Manassas, VA), and the T47D-derived clones DNC8 and LXC5, carrying the dominant negative RARα 403, or the cognate control vector, respectively [9], were cultured in Dulbecco's modified Eagle's medium (DMEM, Invitrogen), supplemented with 5% FBS (Invitrogen, Carlsbad, CA). Cells were all maintained at 37°C in 5% CO_2_ and 85% humidity.

#### Three dimensional (3D)-cultures

HME1 cells and derived clones were grown on reconstituted basement membrane (Matrigel) to induce breast epithelial differentiation into acini-like structures, essentially as described [19]. Briefly, single cells were induced to form acini on chamber slides coated with growth factor-reduced Matrigel (BD Biosciences, San Jose, CA) in medium plus 2% matrigel for 10–15 days. After fixation with 4% paraformaldehyde for 20 min, permeabilization with Phosphate Buffered Saline (PBS) plus 0.1 % Triton X100 for 10 minutes, and blocking with PBS plus 1% BSA, 1% goat serum, 0.05 Tween 20 for 2 h, cells were incubated over night with both an antibody specific for the Golgi apparatus (anti-GM 130, 1:400, BD Biosciences), and an antibody for integrin (anti-CD49f, 1:200, Chemicon, Temecula, CA), followed by detection with goat anti-mouse Alexa Fluor 546 (1:400, Molecular Probes) and goat anti-rat Alexa Fluor 488 (1:400, Molecular Probes, Eugene, OR). Nuclei were counterstained with 300 nM DAPI (Sigma, St. Louis, MO). 30 acini, or more, per each clone were analyzed by confocal microscopy (SP2 Spectral Confocal Microscope, Leica, Wetzlar, DE) to inspect for the presence of a hollow lumen and apicobasal polarization. The morphology observed in 70% or more of the acini was considered to be the prevalent phenotype.

#### Colony formation assay

Exponentially growing cells were seeded at 3×10^2^ cells/well in 6-well plates and allowed to attach for 48 h. After treatment with either 0.1 μM RA or vehicle (ethanol) for 24 h, the medium was replaced with drug-free medium and cells were allowed to grow until the appearance of colonies was observed (10–14 days). Colonies fixed with methanol and stained with Giemsa were analyzed with Image J software (http://rsbweb.nih.gov/ij/) to establish the percentage of growth compared to the non-treated control (colony formation index). Statistical significance was calculated by Student's *t*-test on three independent determinations; p values at least <0.05 were considered as significant.

#### Transient transfections

Wild type (WT) CRABP2 was amplified from pCMV-FLAG-CRABP2 plasmid DNA and CRABP2-KRK was amplified from pSG5-CRABP2-KRK plasmid DNA (kindly provided by Dr. Noa Noy, Case Western Reserve University, Cleveland, OH) [13], by using specific primers (sense: 5′- GCC ACC ATG CCC TTC TCT-3′; antisense: 5′- CTC TCG GAC GTA GAC CCT GG-3′). Both WT-CRABP2 and CRABP2-KRK amplified products were cloned into pcDNA3.1-V5/His TOPO vector (Invitrogen) in frame with the V5-His tag at the 3′ end, and sequenced. HME1 cells grown on glass coverslips in 6-well plates for 24 h were transfected with either pcDNA3.1-WT-CRABP2-V5 or pcDNA3.1-CRABP2-KRK-V5 using Lipofectamine 2000 (Invitrogen) as per manufacturer's instructions. After 24 h cells were treated with either RA (0.1 μM) or vehicle (ethanol) for 30 minutes, fixed in 4% paraformaldehyde for 7 min., permeabilized with PBS plus 0.1% Triton X100 for 5 min., blocked with PBS containing 1% BSA, 1% goat serum and 0.05% Tween 20, and incubated with anti-V5 antibody (1:200) (Invitrogen) as primary antibody for 1 h, rinsed, and detected with an anti-mouse Alexa Fluor 546 secondary antibody (1:400) (Invitrogen). After counterstaining with DAPI, cells were mounted with Vectashield (Vector Laboratories), and analyzed with a Fluorescence microscope (Axioskop, Zeiss).

#### Stable transfections

Cells were transfected with either pcDNA3.1-CRABP2-KRK or the cognate empty vector (EV) by using Lipofectamine 2000, and selected with 1mg/ml G-418 sulfate (Invitrogen). The presence of CRAPB2-KRK mutant was tested both by PCR (sense primer: 5′- GCC ACC ATG CCC TTC TCT- 3′; antisense primer: 5′- CTC TCG GAC GTA GAC CCT GG- 3′) and Western Blotting in independent clones.

#### Stable RNA interference (RNAi)

The sequences CRABP2-A (5′- CTG ACC AAC GAT GGG GAA C-3′), CRABP2-C (5′- GGT TGT CCC TGG ACT TGT C-3′) (Gene Bank NM_001878, nucleotides 477–495, and 9–27 respectively) targeting *CRABP2* mRNA, and the control mock sequence (5′- ACG TAC GTA CGT AGT GGG G-3′), which does not recognize any human mRNA, were cloned into the pSUPER-retro vector according to the manufacturer's instructions (Oligoengine, Seattle, WA). The silencing efficiency of the short hairpin RNAs (shRNAs) produced by these constructs was preliminary tested on exogenous CRABP2 transiently cotransfected with the shRNAs in COS cells as previously described [7]. The pSuper-CRABP2-A, pSuper-CRABP2-C, and pSuper-Mock constructs were stably transfected in HME1 cells by using Lipofectamine Plus (Invitrogen). Single stable clones were selected in puromycin 1 μg/ml, tested for the presence of the correct construct by PCR and sequencing, and further analyzed for the level of endogenous CRABP2 transcript by Real Time RT-PCR.

### Drugs and treatments

All-*trans*-retinoic acid (RA) (Sigma, St. Louis, MO), 5-aza-2′-deoxycitidine (5-Aza) (Sigma), Trichostatin A (TSA) (Sigma), and a specific RARα antagonist ER50891 (a kind gift of Kouichi Kikuchi, Discovery Research Laboratories, Ibaraki, Japan [20]) were dissolved and stored as described previously [9]. Drugs were diluted in MEGM for HME1 cells and derived clones, or DMEM plus 5% charcoal-stripped FBS (Invitrogen) for T47D cells and derived clones. Cells were allowed to attach over night and treated in the dark with different drug combinations as indicated in the Results section. RA-treatment was performed for 24 h for colony formation assays, and for 72 h for transcription assays, adding fresh RA every 24 h. ER50891 treatment was for 24 h, while TSA and 5-Aza treatments were for 72 h.

### Protein Assays

#### SDS PAGE and Western Blot (WB)

Proteins were resolved by SDS-PAGE, blotted on nitrocellulose membrane, and incubated with primary antibodies for GAPDH, RARα (both from Santa Cruz Biotechnology, Santa Cruz, CA), or the V5 tag (Invitrogen).The *in vitro* transcription/translation of CRABP2-V5 was performed using PROTEINscript® II kit (Ambion, Austin, TX) as per manufacturer's instructions. Primary antibodies were detected with appropriate HRP-conjugated secondary antibodies (GE Healthcare, Piscataway, NJ) and followed by ECL (GE Healthcare).

#### Immunoprecipitation (IP)

500 μl cell lysates (lysis buffer: 20 mM Hepes pH 8.0, 5mM EDTA, 150 mM NaCl, 0.5% Triton X100, 0.05% Tween20 plus Complete protease inhibitor cocktail, Roche) were pre-cleared with 40 μl proteinA/proteinG slurry (2/1 by vol.) (Sigma), then incubated over night with anti-CRABP2 antibody. CRABP2-antibody complexes were immunoprecipitated by adding 40 μl proteinA/proteinG slurry (2/1 by vol.), washed, eluted with Laemmli sample buffer and analyzed by Western blot.

### Real time RT-PCR

Total RNA was obtained using Trizol (Invitrogen), treated with DNase I (Ambion, Austin, TX) and retrotranscribed with SuperScript First-Strand Synthesis System (Invitrogen). cDNA was amplified by Real-time RT-PCR on an iCycler (Bio-Rad, Hercules, CA) by using the iQ SYBR Green Supermix (Bio-Rad) and specific primers for *CRABP2* (sense 5′- TTG AGG AGC AGA CTG TGG ATG-3′, antisense 5′- TTG GTC AGT TCT CTG GTC CAC-3′), *RARβ2* (sense: 5′- GAC TGT ATG GAT GTT CTG TCA G-3′; antisense: 5′- ATT TGT CCT GGC AGA CGA AGC A-3′), *CRBP1* (sense: 5′- GGT ACT GGA AGA TGT TGG TC-3′, antisense 5′- CAT CTC TAG GTG CAG CTC AT-3′), *CYP26A1* transcript variants 1 and 2 (sense: 5′- GCA ATC TTC AAC CGA ACT CC-3′; antisense: 5′- CTC CTT AAT AAC ACA CCC GAT G-3′), and *GAPDH* (sense: 5′- GAA GGT GAA GGT CGG AGT C-3′; antisense: 5′- GAA GAT GGT GAT GGG ATT TC-3′). The level of the different transcripts was normalized to the level of the *GAPDH* transcript, and quantified by the threshold cycle Ct method. Statistical significance was calculated by Student's *t*-test on three independent determinations; p values at least <0.05 were considered as significant.

### Quantitative chromatin immunoprecipitation (qChIP)

ChIP was performed using reagents purchased from Upstate (Lake Placid, NY), according to the manufacturer's protocol. Chromatin was immunoprecipitated with antibodies against either acetyl-histone H4 or Polymerase II (both from Upstate), and DNA amplification was carried out by real time-PCR with specific primers encompassing the *RARβ2* RARE [9] (sense: 5′- GGT TCA CCG AAA GTT CAC TCG CAT-3′; antisense: 5′- CAGGCTTGCTCGGCCAATCCA-3′), the *CRBP1* RARE [7] (sense 5′- AGC CTG CAC TGT GAG AAC ACA T-3′, antisense 5′- CCA CCA AGT AGA TGA CAT AAT CA-3′), the proximal *CYP26A1* RARE (P-RARE) (sense 5′- GGA GCT CAG CAC ACC TTG GAT-3′ and antisense 5′- CCA GGT TGC TGC CCA CGT TA-3′), or the distal *CYP26A1* RARE (D-RARE) (sense 5′- GAG TTC ACT CGG ATG TCA CGG-3′ and antisense 5′- CTT TCT GGA CAG CGC CTC CG-3′). The relative enrichment of immunoprecipitated DNA was calculated by normalizing the PCR signals of the samples to both the input and the no antibody controls. Amplification of the *GAPDH* promoter region (sense: 5′- GGT GCG TGC CCA GTT GAA CCA-3′; antisense: 5′- AAA GAA GAT GCG GCT GAC TGT CGA A-3′) was used as an internal control. Statistical significance was calculated by Student's t-test on three independent determinations; p values at least <0.05 were considered as significant.

### DNA methylation analysis

Genomic DNA was extracted with DNAzol (Invitrogen) according to the manufacturer's instructions. DNA was modified by sodium bisulfite treatment as previously described [21] and used for either bisulfite sequencing or quantitative Methylation Specific PCR (qMSP) by real time PCR with iQ SYBR Green Supermix (Bio-Rad, Hercules, CA) on an iCycler (Bio-Rad). For *RARβ2* qMSP we used a previously described primer set (M4 sense 5′- GTC GAG AAC GCG AGC GAT TC-3′ and M4 antisense 5′- CGA CCA ATC CAA CCG AAA CG-3′) [9], [11]. For *CRBP1* qMSP we used the following primers, specifically amplifying two methylated CRBP1 regions: M1 sense 5′- CGT TTT TGC GTT CGT TTT CGT TAA GC-3′ and AS1 antisense 5′- AAA TAA CTA AAA CCA ATT AAC CAC AAA-3′; M2 sense 5′- CGT TGC GTT TTG GGC GTT TCG TC-3′ and AS2 antisense 5′- CAC CAA ACC ACA ACT CAC CAA A-3′. For CYP26A1, the 5′ region of the gene was first analyzed for the presence of canonical CpG Islands by using CpG Island Searcher (http://cpgislands.usc.edu/). For bisulfite sequencing of the CpG island containing the proximal RARE, bisulfite modified DNA was amplified by nested PCR with Platinum Taq (Invitrogen). The first PCR round was performed with the following primers: P772 sense 5′- TAT TAY GTG GAA GAG AGT TTA T-3′ and P773 antisense 5′- ACT TCA ACA AAA ACC CAA AAC-3′. The second PCR round was performed with the following primers: P776 sense 5′GAA GGT TAG AGT TTG GAA TTT-3′ and P775 antisense 5′- CCT ACA ATA CCA TCT ACA AAA-3′. The PCR product was gel-purified and sequenced. For qMSP of the proximal CpG island, bisulfite modified DNA was amplified by nested PCR. The first PCR round was performed as described for bisulfite sequencing. The second round was performed by real time PCR with iQ SYBR Green Supermix (Bio-Rad) in combination with primers specific for methylated CpGs (P764 sense 5′- TCG GCG CGG AAT AAA CGG T-3′ and P765 antisense 5′- CGC GCC GCG ACC TCC CGC GC-3′). The PCR signal from the M alleles was normalized to the signal from a control *CYP26A1* region amplified by using primers that do not recognize any CpG (P774 sense: 5′- TTA GTG AAG GTT GTT TTG GGT-3′ and 5′- AAT ACA AAT CCC AAA ACT TAA-3′). Statistical significance was calculated by Student's t-test on three independent determinations; p values at least <0.05 were considered as significant.

## Supporting Information

Figure S1 Development of CRABP2 knock down clones. (A) Scheme of the short hairpin (sh) RNA sequences cloned into the pSUPER vector and subsequently used for HME1 stable transfection (left). Transient co-transfection experiments followed by WB analysis showing that the CRABP2-targeting sequences CRABP2-A and CRABP2-C, but not the scrambled sequence Mock, can effectively decrease the protein level of exogenous CRABP2 (right). (C) Sequencing analyses showing that the stable clones Si-CRABP2-A6, Si-CRABP2-C6 and Mock-13 contain the correct p-SUPER construct.
(0.76 MB DOC)

Figure S2 CYP26A1 downregulation in human cells with an impaired RA-RARα signaling is marked by epigenetic chromatin changes. (A) Hampering RA availability at RARα by treatment with the RARα-specific antagonist ER50891 can significantly antagonize RA-induced transcription of both RARβ2 (top) and CYP26A1 (bottom) in human cells (T47D). (B) T47D cells stably expressing a RARα dominant negative protein (DNC8), and cognate control cells (LXC5), are CRABP2-positive (top). (C) Impairment of RARα function in DNC8 cells significantly downregulates RA-induced transcription of both RARβ2 (left) and CYP26A1 (right) relative to control LXC5 cells. (D) CYP26A1 transcriptional repression in DNC8 cells is associated with significant histone H4 hypoacetylation, unresponsive to RA, at the CYP26A1 regions encompassing either the distal RARE (D-RARE), or the proximal RARE (P-RARE). (E) Treatment of DNC8 cells with either TSA (24 h), or 5- Aza (72 h) can restore RA-induced transcription from both RARβ2 (left) and CYP26A1 (right).
(3.50 MB TIF)

Figure S3 In silico identification of human CYP26A1 CpG islands. Analysis of the CYP26A1 5′ regulatory regions by using CpG Island Searcher identifies two CpG islands: one containing the distal RARE (D-RARE), from −2086 to −1502, and one containing the proximal RARE (P-RARE), from −375 to +2239.
(9.31 MB TIF)
