# Supplementary figures and images for: Derangement of a Factor Upstream of RARα Triggers the Repression of a Pleiotropic Epigenetic Network
Source: PLoS One. 2009 Feb 4;4(2):e4305. doi: 10.1371/journal.pone.0004305 (PMC2627936; doi:10.1371/journal.pone.0004305)

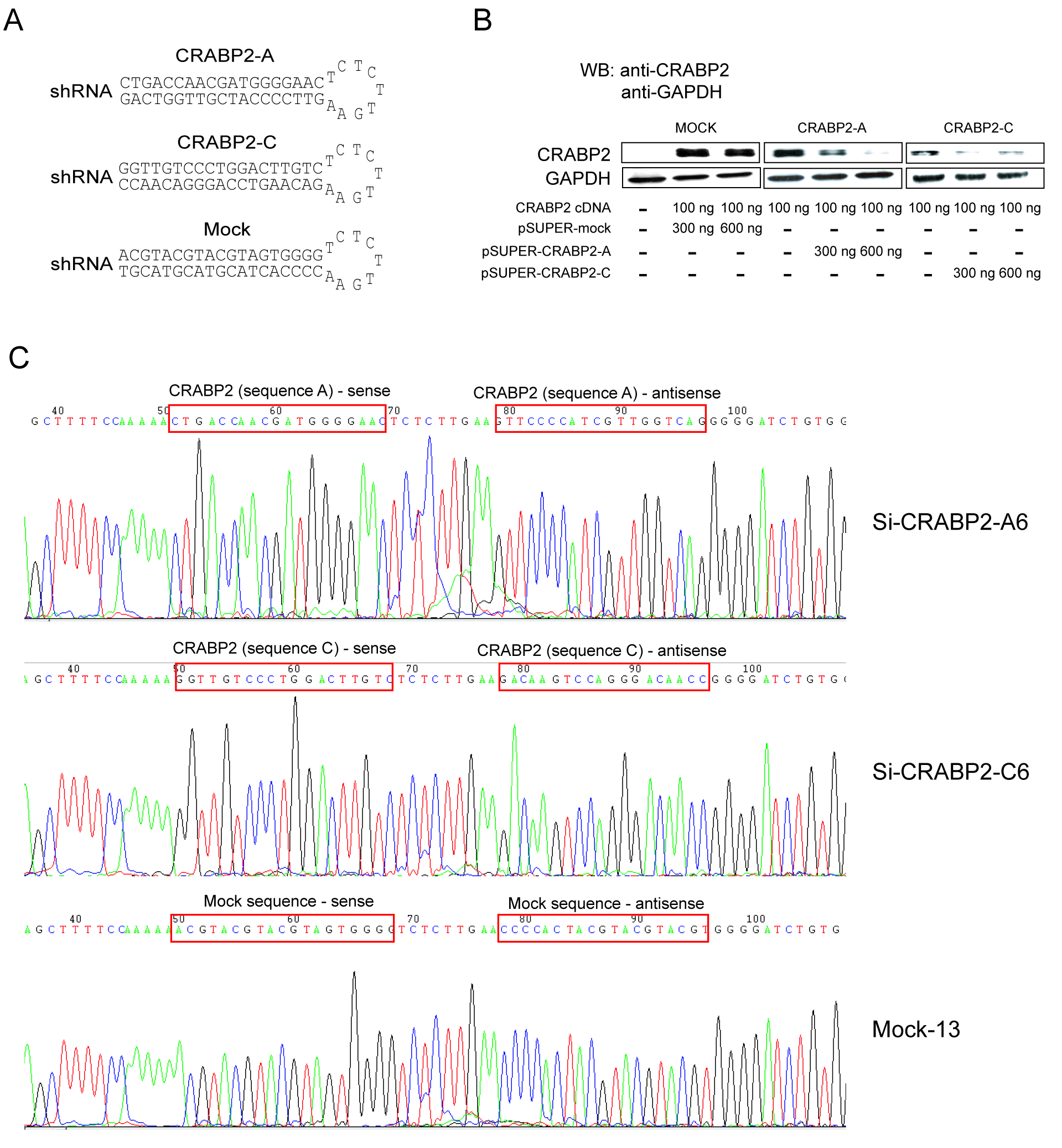

Supplement: Figure S1 — Development of CRABP2 knock down clones. (A) Scheme of the short hairpin (sh) RNA sequences cloned into the pSUPER vector and subsequently used for HME1 stable transfection (left). Transient co-transfection experiments followed by WB analysis showing that the CRABP2-targeting sequences CRABP2-A and CRABP2-C, but not the scrambled sequence Mock, can effectively decrease the protein level of exogenous CRABP2 (right). (C) Sequencing analyses showing that the stable clones Si-CRABP2-A6, Si-CRABP2-C6 and Mock-13 contain the correct p-SUPER construct. (0.76 MB DOC) [file pone.0004305.s001.doc]

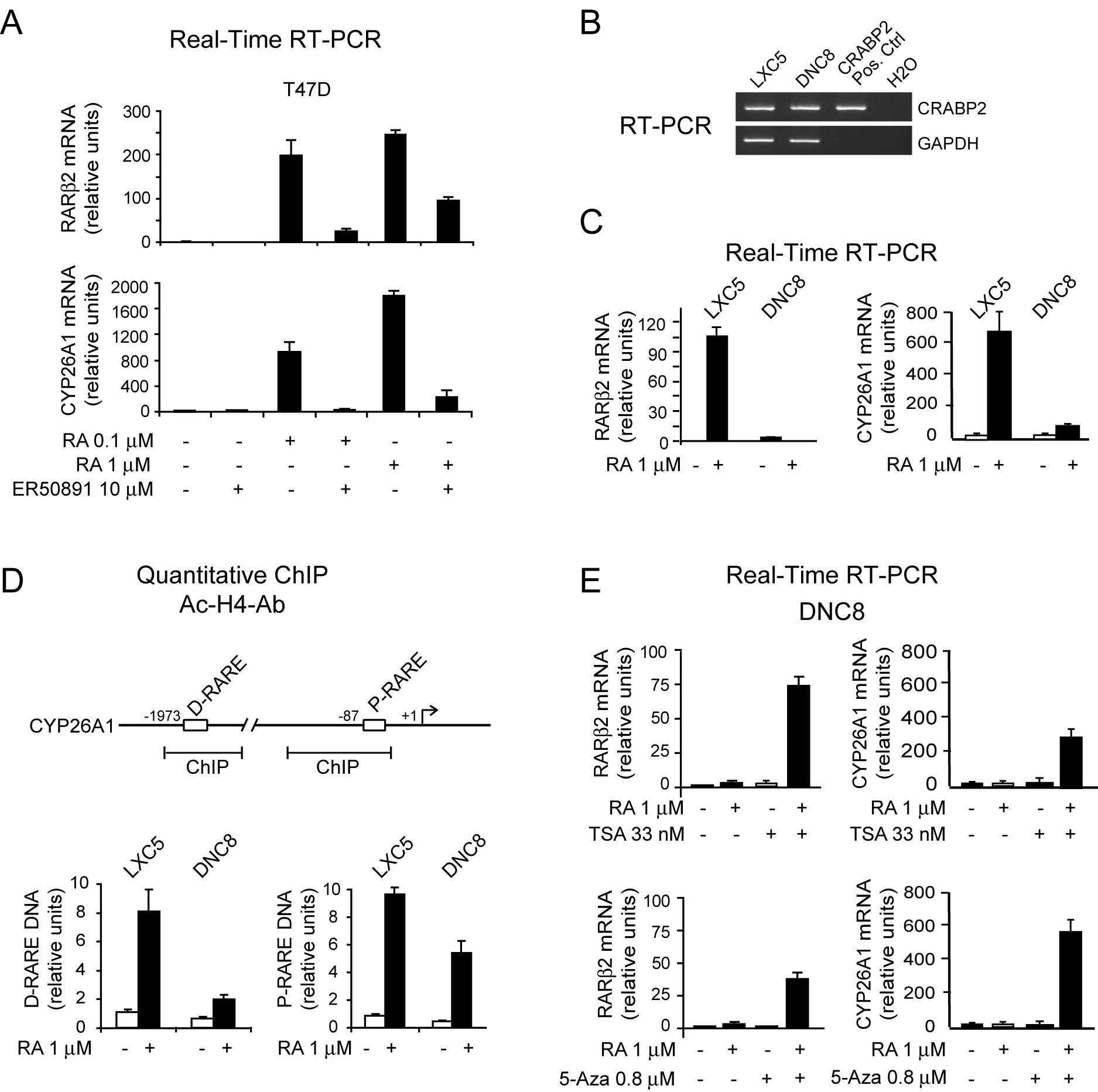

Supplement: Figure S2 — CYP26A1 downregulation in human cells with an impaired RA-RARα signaling is marked by epigenetic chromatin changes. (A) Hampering RA availability at RARα by treatment with the RARα-specific antagonist ER50891 can significantly antagonize RA-induced transcription of both RARβ2 (top) and CYP26A1 (bottom) in human cells (T47D). (B) T47D cells stably expressing a RARα dominant negative protein (DNC8), and cognate control cells (LXC5), are CRABP2-positive (top). (C) Impairment of RARα function in DNC8 cells significantly downregulates RA-induced transcription of both RARβ2 (left) and CYP26A1 (right) relative to control LXC5 cells. (D) CYP26A1 transcriptional repression in DNC8 cells is associated with significant histone H4 hypoacetylation, unresponsive to RA, at the CYP26A1 regions encompassing either the distal RARE (D-RARE), or the proximal RARE (P-RARE). (E) Treatment of DNC8 cells with either TSA (24 h), or 5- Aza (72 h) can restore RA-induced transcription from both RARβ2 (left) and CYP26A1 (right). (3.50 MB TIF) [file pone.0004305.s002.tif]

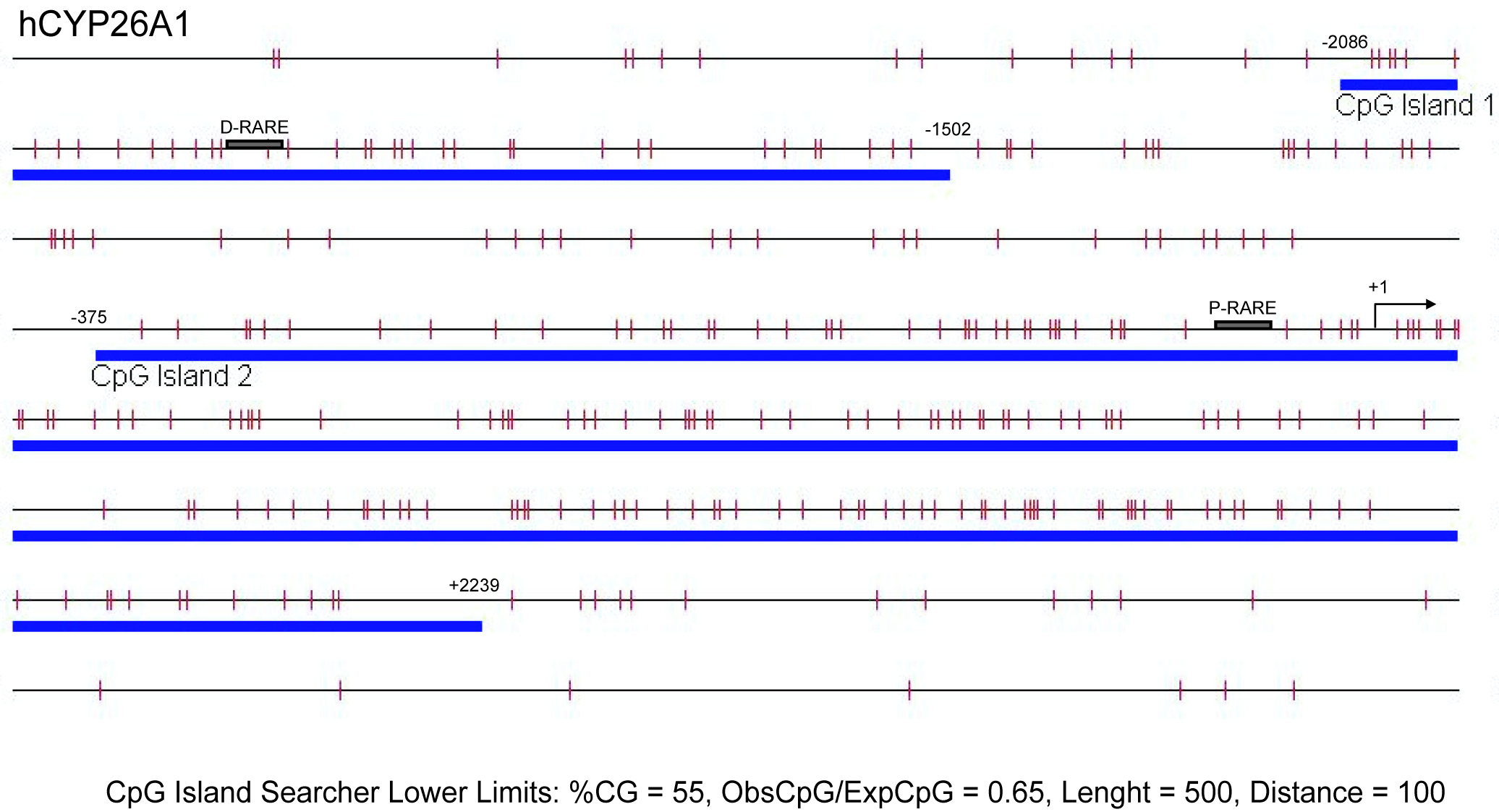

Supplement: Figure S3 — In silico identification of human CYP26A1 CpG islands. Analysis of the CYP26A1 5′ regulatory regions by using CpG Island Searcher identifies two CpG islands: one containing the distal RARE (D-RARE), from −2086 to −1502, and one containing the proximal RARE (P-RARE), from −375 to +2239. (9.31 MB TIF) [file pone.0004305.s003.tif]
